# Exploring Overnutrition, Overweight, and Obesity in the Hospital Setting—A Point Prevalence Study

**DOI:** 10.3390/nu15102315

**Published:** 2023-05-15

**Authors:** Andrea Elliott, Simone Gibson, Judy Bauer, Anna Cardamis, Zoe Davidson

**Affiliations:** 1 Department Dietetics, Nutrition and Food Monash University, Notting Hill, VIC 3168, Australia; andrea.elliott@monash.edu (A.E.); simone.gibson@monash.edu (S.G.); judy.bauer@monash.edu (J.B.); 2 Nutrition and Dietetics Department, Eastern Health, Box Hill, VIC 3128, Australia

**Keywords:** overnutrition, obesity, malnutrition, hospital nutrition care

## Abstract

Malnutrition is an international healthcare concern associated with poor patient outcomes, increased length of stay, and healthcare costs. Although malnutrition includes both under and overnutrition, there is a large body of evidence that describes the impacts of undernutrition with limited data on overnutrition in hospitalized patients. Obesity itself is a modifiable risk factor associated with hospital-associated complications. However, there is limited reporting of the prevalence of obesity in hospitals. This one-day cross-sectional study (*n* = 513) captures the prevalence of both under and overnutrition in a hospitalized population and explores dietetic care provided compared to the Nutrition Care Process Model for hospitalized patients who have obesity. The main findings were: (1) the largest proportion of patients were in the overweight and obese classifications (57.3%, *n* = 294/513); 5.3% of these patients had severe obesity (class III); (2) patients who were overweight and obese had lower malnutrition risk profiles as well as the prevalence of malnutrition; (3) 24.1% of patients who had obesity (*n* = 34/141) were receiving dietetic intervention; (4) 70.6% (*n* = 24/34) did not have a nutrition diagnosis that followed the Nutrition Care Process Model. Study results provide valuable clinical insight into the prevalence of overnutrition and opportunities to improve nutrition care for this vulnerable patient group.

## 1. Introduction

Malnutrition in hospitals is a global concern and is associated with increased cost, length of stay, and morbidity and mortality for patients [1,2,3]. Internationally, the prevalence of malnutrition in hospitals has been widely reported, with models of nutrition care supporting best practices in the prevention and management of malnutrition [4,5,6]. Recently, there has been a change to clinical governance and quality systems in Australian hospitals, with malnutrition risk screening integrated as part of the risk assessment for all patients admitted to hospital [7] and nutrition care focused on patients who are identified as having undernutrition and the management of malnutrition in patients who are under a healthy weight [8,9]. Although malnutrition refers to all deviations from adequate and optimal nutritional status, comprising both undernutrition and overnutrition [9,10], there is very little data on the prevalence of overweight and obesity in hospitalized patients.

The impact of overweight and obesity as a risk factor for chronic diseases is widely known, as is the impact on the burden of disease. In 2015, overweight and obesity contributed 8.4% to the burden of diseases, second only to tobacco use (9.3%). Obesity is associated with both greater duration and cost of hospitalization [11], with significant differences in the average length of stay found for almost all medical and surgical specialties for patients who have obesity [2,12,13]. Patients who have obesity have higher complications post-surgery [14,15], are at risk of skin breakdown and impaired wound healing [16], increased rates of infection and sepsis [11,15,17,18], have a greater chance of admission to the intensive care unit [17], and an increased risk of morbidity [18] and mortality [11,17]. A substantial proportion of hospital admissions and costs are attributed to overweight and obesity, with 13% of total admissions or 1 in 8 admissions and 17% of total costs [19]. The most recent Australian National Health Survey (2017–2018) found that 67% of adults were overweight or obese (12.5 million people), an increase from 63.4% in 2014–2015 [20]. As obesity is a modifiable risk factor for ill health and chronic disease, data on the proportions of hospitalized patients who are classified as overweight and obese will assist in better designing and planning effective models of care and services that focus on the full spectrum of malnutrition.

This study aimed to provide a current snapshot of the demographics and body mass index (BMI) of the patient cohort of a large, metropolitan, multicentered tertiary health service in Victoria, Australia, to capture the prevalence of both under and overnutrition; and compare this to current published Australian and state population norms. The secondary aims of this study were to explore the relationship between BMI, clinical characteristics, and other independent factors; and to identify the nutrition diagnosis, nutrition care planning, and dietary intervention provided to hospitalized patients who were classified by BMI as having obesity.

## 2. Materials and Methods

This study was a multi-site cross-sectional audit of inpatients across a single health service comprising six hospital sites and 1507 beds at Eastern Health in Melbourne, Australia, in 2021. In 2018–2019, the health service had 1,350,629 episodes of care and 169,465 emergency presentations [21]. This study was submitted to the Eastern Health Research and Ethics Office for ethics approval and was considered to have met the criteria for an audit or quality assurance activity [22] and, therefore, registered as a quality assurance activity, reference no: QA20-123. The project was also registered by the Monash University Human Research Ethics Committee, Project no: 37575.

Data were collected on a single day in October 2021. All hospital inpatients ≥ 18 years of age admitted for more than a two-night stay during the data collection period were eligible for inclusion in the study. All acute, sub-acute, mental health, and transition care program inpatient wards were included in data collection at all sites. Across all sites, patients were excluded if they were deemed an infection risk, medically unstable, or in the final stages of palliation; had significant swelling or had undergone medical interventions that may impact body weight and body mass index, including edema and ascites; maternity patients; mental health patients, or patients with low levels of consciousness deemed unable to participate.

Patients with an amputation were included in the study, with body weights adjusted before analysis. Patients with an amputation had the weight of their missing body part estimated via a body part percentage algorithm [23], and this weight was added to their measured overall body weight. The body weight of patients with acute kidney injury or chronic kidney disease requiring renal replacement therapy was measured post-dialysis session.

Data collected included age, sex, admitted medical unit/ specialty, height (cm), and weight (kg). Height and weight measurements documented in patient medical records were used where available (up to 7 days prior to audit day if the patient had admission >7 days), otherwise directly measured with a stadiometer and calibrated scales, respectively, on the day of the audit. Where this was not possible, height was estimated by alternative measures, i.e., ulna length or verbally reported by the patient, and weight was reported by the patient or estimated by the data collector. BMI was defined and categorized by WHO criteria (BMI > 30.0 kg/m^2^), with obese class I, II, and III all included in the overall definition of obesity [24].

Data collected about nutrition care for all patients surveyed included malnutrition risk, malnutrition diagnosis, and care received by a dietitian. Malnutrition risk was determined using a validated and reliable malnutrition risk screening tool, the Malnutrition Screening Tool (MST), where a score of 0–1 indicates not being at risk of malnutrition [1,25]. Nutrition care received by a dietitian during admission included identification, documentation, and treatment of malnutrition. A diagnosis of malnutrition was determined where the patient matched the following criteria using the ICD-10-AM definition of BMI < 18.5 kg/m^2^ or unintentional loss of weight ≥ 5 percent with evidence of suboptimal intake resulting in subcutaneous fat and/or muscle wasting [26]. Malnutrition diagnosis was validated by documentation in the medical history by a dietitian or doctor (as per coding requirements). When patients who were classified as having obesity were identified as receiving dietetic assessment and intervention, the medical record was further interrogated to determine nutrition diagnosis, including documentation of a nutrition diagnostic statement using Nutrition Care Process Terminology (NCPT) and intervention that followed the Nutrition Care Process (NCP) Model [27]. Nutrition diagnoses were analyzed and coded to the three diagnosis domains of NCPT: clinical, intake, and behavior [28].

A data collection tool was developed specifically for the audit and piloted to ensure reliability. Study data were collected and managed using an electronic data capture tool hosted at Eastern Health. REDCap (Research Electronic Data Capture) is a secure, web-based software platform designed to support data capture for research studies, providing (1) an intuitive interface for validated data capture; (2) audit trails for tracking data manipulation and export procedures; (3) automated export procedures for seamless data downloads to common statistical packages; and (4) procedures for data integration and interoperability with external sources [29,30]. A paper version of the survey tool was available where required. All sites utilized dietitians and final-year undergraduate dietetic university students who were trained and supervised in pairs to collect data for all patients.

### Statistical Analysis

Statistical analysis was conducted using SPSS, Version 28.0 (SPSS Inc., Chicago, IL, USA). For all analyses, significance was considered to be obtained when *p* ≤ 0.05. Results are displayed as mean and standard deviation (SD) for normally distributed data and median and interquartile range (IQR) for non-normally distributed variables. The distribution of each variable was examined visually using histograms. The normality of data distribution was tested using the Shapiro–Wilks Test.

Descriptive analyses of the physical and clinical characteristics of the cohort were performed. Analyses of means and standard deviations or medians and interquartile ranges were conducted based on the distribution of the data. The Chi-square test was used to explore the relationship between BMI and categorical variables, and the Kruskal–Wallis H test was used for BMI and continuous variables. Cramer’s V was used to describe the strength of association for Chi-square analysis, with an effect size > 0.29 considered to be a strong relationship [31].

Multiple regression analysis was used to explore the relationship between BMI, clinical characteristics, and other independent variables. Clinical factors reasonably expected to relate to BMI were selected for the model. Dietetic intervention was included as this was determined to be a clinically relevant factor related to the identification and management of malnutrition and weight management. Clinical characteristics entered into the model included sex; other variables were nutrition risk screen completion (yes/no), nutrition risk score, diagnosis of malnutrition (yes/no), and dietetic assessment and intervention (yes/no). Standard multiple regression, in which all independent variables are entered into the equation simultaneously, was used in the analysis. Before proceeding with the model, the following assumptions were examined when performing the multiple regression: multicollinearity, normality and linearity residuals, homoscedasticity, and the presence of outliers [31]. No other variables were entered into the regression model.

To assess the model, we considered R^2^ of the overall model, the significance of independent variables as well as the contribution of individual variables to the model determined from the partial correlations.

## 3. Results

### 3.1. Prevalence of Overweight and Obesity

There were complete data for 513 patients across seven hospital sites in acute, subacute, and transition care programs on the day of the audit (80.7% eligible patients). Measured heights and weights were available for 94% of the total study sample. The largest proportion of patients were admitted under the acute program (64.3%). 

Table 1 describes the general and nutritional characteristics of the study sample according to the BMI category. The median age was 73 years (IRQ: 59–83 years), and there were approximately equal proportions of males and females (females 49.7%) in the sample. The median BMI across the sample was 27.7 kg/m^2^ (IQR 22.2–30.4 kg/m^2^). The largest proportion of patients were in the overweight and obese classifications (57.3%); 5.3% of these patients had severe obesity (class III).

Malnutrition risk profiles were available for 70.6% (*n* = 362) of the sample, with 68.8% (*n*=249) of patients with a completed nutrition risk tool scoring low risk on the nutrition risk screening using the MST. A higher proportion of patients in the underweight and healthy weight classifications scored at risk (59.2%, *n* = 67). The prevalence of malnutrition for the total sample was 16.8%, with the prevalence of malnutrition in the underweight and healthy weight classifications higher than the total sample at 70% (*n* = 21/30) and 24.7% (*n* = 47/189), respectively. The patients that were overweight and obese had lower malnutrition risk profiles as well as the prevalence of malnutrition, with 14 patients (9.2%) that were overweight and four patients (2.8%) that had obesity diagnosed with malnutrition.

The Chi-square test found a significant association between weight classification and sex (*p* = 0.002, Cramer’s V = 0.193), malnutrition diagnosis (*p* < 0.001, Cramer’s V = 0.433), and dietetic intervention (*p* < 0.001, Cramer’s V = 0 237. Malnutrition diagnosis had the strongest association. Kruskal–Wallis H test revealed a statistical difference in age between all weight classification groups χ^2^ (5, *n* = 513) = 44.48, *p* < 0.001. The underweight group had a significantly higher median age of 89 years (IQR: 74–94) than the obese class III group’s median age of 64 years (*p* < 0.001).

The cohort was then categorized into the clinical program (Table 2). The largest proportion of patients were admitted to the acute inpatient program (64.3%, *n* = 330). The median age was 70 years (IRQ: 55–81 years), and there were fewer females (44.5%) than males admitted. The median BMI across the patient surveyed in the acute program was 26.7 kg/m^2^ (IQR: 23.1–31 kg/m^2^). A larger proportion of patients were overweight and obese (62.7%) in the acute inpatient program than in the overall sample, with 4.8% of these patients having severe obesity (class III). In the patients admitted under the acute program, the Chi-square test found no significant association with sex, *p* = 0.053. The Kruskal–Wallis H Test revealed a statistical difference in BMI χ^2^ (5, *n*= 330) = 303.995, *p* < 0.001 but not age. The Chi-square test found no significant association with sex, *p* = 0.053, Cramer’s V = 0.182. In the subacute group, the Chi-square test found a significant but weak association with sex, *p*= 0.047, Cramer’s V = 0.271; Kruskal–Wallis H test revealed a statistical difference in age χ^2^ (5, *n* = 153) = 30.34, *p* < 0.001. The underweight group had a significantly higher median age of 93 years (IQR: 89–96). In the transition care program, there was no relationship between age and sex, and BMI classification.

### 3.2. Nutrition Care Characteristics

**Figure 1:**
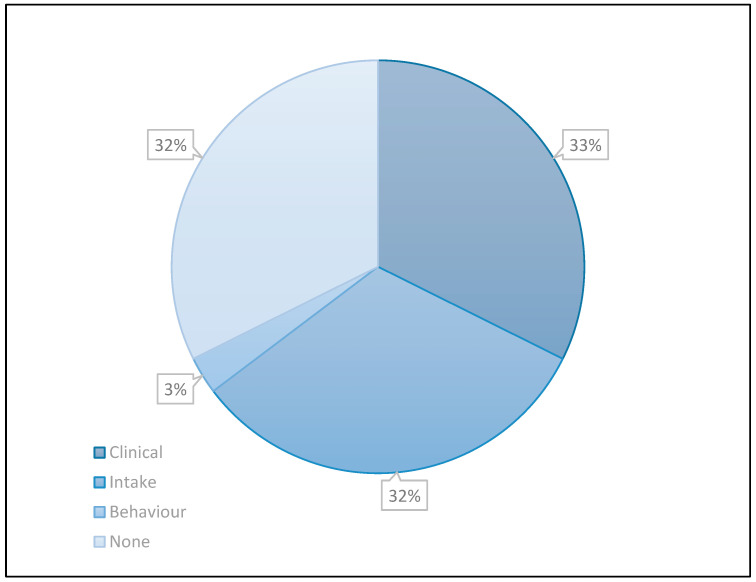
Classification of nutrition diagnosis by diagnosis domains of patients who had obesity and were receiving dietetic care.

A total of 37.3% (*n* = 191) patients were receiving care from a dietitian. The greatest proportion of patients receiving nutrition care was in the underweight and healthy weight classifications (45.2%). Of the patients that had obesity and severe obesity, 24.1% (*n* = 34/141) were receiving dietetic intervention. Ten of these patients had a nutrition diagnostic statement that followed Nutrition Care Process (NCP) terminology in a structured sentence that included nutrition diagnosis: Problem, Etiology, and Signs and Symptoms. For a further 13 patients, a nutritional problem was identified, but without a diagnostic statement. Nutrition diagnoses could be categorized into the following classifications: Clinical, Intake, and Behavior, as shown in Figure 1. The majority of nutritional problems could be categorized as clinical or intake. Seven patients (20.6%) in the clinical category were identified as well-nourished in the context of having obesity (Table 3).

The reasons for dietary intervention varied for patients who had obesity or severe obesity. Four patients received dietetic care with the goal of resolving undernutrition. Four patients were receiving dietary intervention, counseling, and support related to weight management and had documentary evidence of goal setting with the patient. Further, these same patients had a nutrition care plan developed to support nutrition-related behavior change for weight management documented in the medical record.

### 3.3. Comparison to Population Data

**Figure 2:**
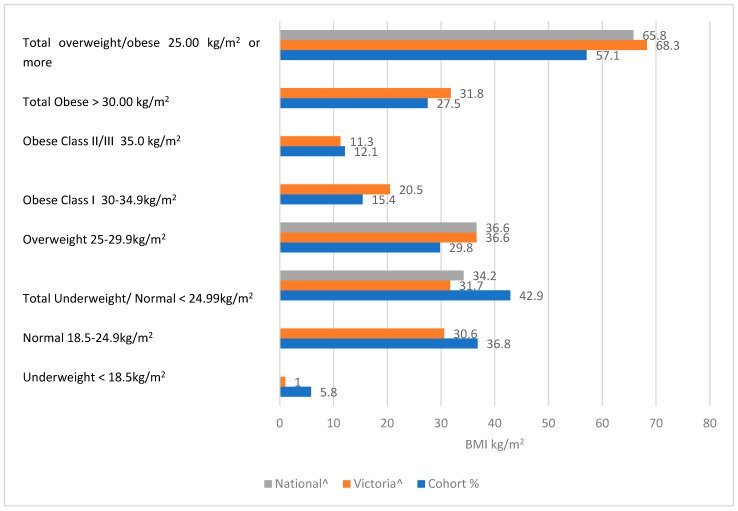
Comparison of study sample vs. Victoria and Australia population norms by classification of weight status by BMI category, adults > 18 years. Adapted with permission from Australian Institute of Health and Welfare material Ref. [20]. 2022, Australian Institute of Health and Welfare Copyright ©.

Comparison with both Victorian and National population norms for 2018–2019 [20] are provided in Figure 2, with data reflecting higher proportions of the hospital population in the under-weight and obese class II and III classifications and lower proportions in the overweight and obese class I categories when compared with Victorian and National population data (Refer to Appendix A). A Chi-square Goodness of Fit test indicated there was a significant difference in the proportions of this sample of hospitalized patients in the underweight, normal weight, and obese II/III classifications than the Victorian population χ^2^ (5, *n* = 513) = 140.27, *p* < 0.001.

### 3.4. Regression Analysis

Standard multiple regression was used to explore relationships between BMI and other factors (Table 4). The overall regression model predicted 16.7% of the variation in BMI (R^2^ = 0.167, F(7, 505)) = 15.617, *p* < 0.001). Independent variables in the model significantly associated with BMI included age, sex, nutrition risk score, and malnutrition diagnosis. Of these variables, malnutrition diagnosis made the largest contribution to the model, explaining 5.15% of the variance in BMI. Specifically, an affirmative malnutrition diagnosis was associated with a lower BMI.

## 4. Discussion

This point prevalence study documented the BMI of patients in hospital, finding over half of the patients were overweight and obese (57.3%). This was slightly less than state and national population data; however, in comparison, higher proportions of the hospital population were in obese class II and III classifications. In our study population, 27.5% of inpatients had obesity, with the majority of these patients receiving acute medical care. This prevalence of obesity is similar to a 2020 study (*n* = 1327) in Queensland which found 30% of inpatients to have obesity; we also found a similar prevalence of severe obesity of 5.8%, compared to 7% [32]. Our findings are in contrast to a 2015 study (*n* = 416) in Western Australia that found a 19% prevalence of obesity in a hospitalized population, with 4% classified with severe obesity [33]. All three studies used comparable methodologies. While the increasing global trend in the prevalence of obesity is well documented [34], internationally, there is limited exploration of the prevalence across hospitalized populations. Instead, studies have focused on the prevalence of obesity in specific patient cohorts, for example, general medical (30%) [35], cardiology (31.5%) [36], and cardiac surgery (39.2%) [15], with these studies finding a similar prevalence.

This study identified a similar proportion of patients classified in the underweight (5.8%) and severely obese (5.3%) categories. However, significantly more of the patients in the underweight category were identified as nutrition risk (36.6% vs. 11.1%) and had a diagnosis of malnutrition (70% vs. 3.7%): Multiple regression analysis affirmed that a malnutrition diagnosis was associated with a lower BMI. More patients who were beneath their healthy weight range received dietetic care than patients having obesity whilst an inpatient (73.3% vs. 29.6%). With the focus of routine nutrition risk screening tools on identifying underweight and undernutrition in adults and dietetic care concentrated on nutrition deterioration to prevent hospital-acquired malnutrition [4], dietitians do not consistently identify overweight and obesity as a nutrition problem in the acute setting; patients in obese weight classifications are therefore an underserviced population with nutritional needs that would benefit dietetic assessment and intervention. In our study, only 29.6% of patients having obesity were seen by a dietitian, with the majority of patients, 70.4%, not receiving dietetic care. There has been an evolution of malnutrition diagnostic criteria to move beyond the major malnutrition concept for adults, which is described by BMI < 18.5 kg/m^2^, to include all BMI categories as well as phenotype and etiology. There has been a push to move from the current International Classifications of Diseases (ICD-10AM) coding definition for malnutrition to a more clinically relevant and contemporary definition of malnutrition in adults as an amendment for ICD-11 [37], encompassing obese weight classifications and overnutrition [9].

In addition to malnutrition, ensuring patients who are overweight and obese receive timely nutrition care could potentially prevent other hospital-related complications such as pressure injuries (PIs) [38,39]. Patients who have severe obesity are at risk of developing a PI due to poor mobility, vascularity, moisture, and skin integrity, especially if bed-bound [39]. Severe obesity is a significant and independent risk factor for developing a PI; however, the odds of developing a PI for a person who has overnutrition and severe obesity are substantially increased, with a prevalence of 4.8% in patients having obesity and 12% in patients being morbidly obese [39], and patients who have obesity are at risk of poor or delayed wound healing [38,40]. There are also significant impacts on length of stay and health care costs associated with PIs, where Ngheim et al. found PIs to be associated with 1.53 million bed-days of excess length of stay across Australian public hospitals [40].

Although identification and management of undernutrition is an issue in hospitals, for many hospitalized patients addressing the nutrition problem of obesity will benefit health outcomes. Studies have found having morbid obesity leads to worse health outcomes, including longer lengths of hospital stays [12,13,15,17] and increased risk of hospital complications [17,18,41], including infection post-surgery [15]. In addition, patients who have obesity are more likely to be admitted to the Intensive Care Unit post-trauma [17]. In the critical care setting, the provision of adequate nutrition support to patients who have severe obesity is challenging [42], and patients who have obesity appear to have lower mortality but an increased risk of complications in several organ systems [18]. Obesity is also an independent risk factor for hospital readmission following surgery, such as total joint arthroplasty [43], cardiac surgery [41], and exacerbation of chronic health conditions [38,44], including mental health conditions [45].

Managing the nutritional needs of people with obesity is challenging, and it is essential to identify the underlying causes in order to select strategies that will support people to lose weight. In clinical practice, Dietitians utilize the Nutrition Care Process (NCP) and NCP terminology (NCPT). The NCP is supported globally by the International Confederation of Dietetic Associations and national dietetic associations [46,47,48,49]. The NCPT is a standardized language that supports the four steps of the NCP: Nutrition Assessment and Reassessment, Nutrition Diagnosis, Nutrition Intervention, and Nutrition Monitoring and Evaluation [27,49,50]. The inclusion of the diagnosis step is critical as it refers to a nutrition diagnosis rather than a medical diagnosis. Our study found that 29% of patients (*n* = 10) who had obesity and were receiving dietetic care had a complete nutrition diagnostic statement using NCPT [27]; 32% of the patients who had obesity (*n* = 11) did not have a nutrition diagnosis documented. A further 21% (*n* = 7) had an inaccurate nutrition diagnosis where the nutrition problem was identified as being well nourished. The absence of nutrition diagnosis and inaccurate PES statements indicate dietitians may have difficulty understanding obesity as a nutritional problem. Selecting an appropriate nutrition diagnosis can be a complex process requiring both critical thinking and clinical reasoning and involves the interpreting, organizing, and grouping of data to enable the selection of the diagnosis [49]. In a review that describes the dietary risk factors associated with childhood obesity, Kim and Lim [50] propose an adoptable nutrition diagnosis of NCP components while using NCPT for childhood obesity. This customized approach to nutrition diagnosis could be used by dietitians caring for adults who have obesity. The NCP is locally supported by the Dietetics professional association Dietitians Australia, and there is evidence that local knowledge, attitudes, and adoption of the NCPT have progressed since its implementation in 2014 [49]. However, the current application of NCPT in the acute care setting may constrain nutrition diagnosis, with the focus of nutrition care on undernutrition. With 11 patients who had obesity in our study that did not have a nutrition diagnosis documented, there appears to be further opportunity for learning and development of dietitian clinical decision-making for determining the nutrition diagnosis for people who have obesity to enable the design of effective person-centered nutrition care [51] to support overnutrition and weight management.

Effective treatment and management of overweight and obesity require a comprehensive, personalized plan supported by a multidisciplinary team [52], of which dietitians are integral members. Despite the significant proportions of inpatients who are overweight and obese in the acute care setting, treatment is largely limited to surgical intervention [53], as primary care is the preferred setting for first-line treatments [54] and behavioral weight management interventions [55]. There is a reluctance by health professionals to raise obesity and weight as an issue with patients [56], and clinicians, including dietitians [57,58,59], may hold implicit negative attitudes and beliefs toward patients who are overweight or obese [56,60]. There are also challenges in care provision. Patients who have obesity may need specialist support from treating teams, from continence specialists, respiratory physicians, wound care nurses, clinical psychology, and occupational health and safety representatives [60]. Health services need to ensure they have appropriate governance frameworks, leadership, policy, clinical pathways, and equipment [56,60,61] in place that support the best care of patients who have obesity as well as treating teams that are empathetic and are able to provide personalized care [56,60,62]. As a starting point, this may require an exploration of nutrition risks identified for patients in the hospital setting. As well as undernutrition, current quality care standards for hospitals focus on nutrition risks related to dehydration, dysphagia, special dietary needs, food intolerance, and allergy [7]. The standards do not include obesity and being overweight, although this is a modifiable risk factor that can be effectively managed by the multidisciplinary team. To address the health implications associated with obesity, nutrition risk screening tools may need refinement. Further, we may need to understand more about the views of health professionals, and specifically dietitians, about people who have obesity and address challenges in identifying and diagnosing overnutrition as a nutrition problem; to effectively care for these patients in the acute care setting.

This study found the prevalence of overweight and obesity in hospitalized patients to be comparable to current national and state population data [20], with the prevalence of people who have severe obesity in hospital greater than those in the population (12.1% vs. 11.3%). While this is not statistically significant, it has considerable clinical meaning. Over the last 40 years, the global prevalence of obesity has increased. Implementation of policy and healthcare interventions has stopped the progress of childhood obesity in some countries, but no country has decreased the obesity epidemic across its population [63,64]. Without a considerable effort to reduce the prevalence of obesity, there will be an ongoing impact on the healthcare system, with overweight and obesity contributing more significantly to the burden of diseases, hospital admissions, and healthcare costs.

### 4.1. Strengths and Limitations

The strengths of this study are this is the first study known to the authors to explore obesity, overnutrition, nutrition care, and dietetic practice in the hospital setting. The large sample size, response rate, and heterogeneous nature of the study of the population across a multisite health service inclusive of a number of clinical programs and medical specialties enhance the transferability of data to a variety of other health services, hospitals, or care settings. Standard training was conducted for all data collectors to ensure the understanding of all data elements, strengthening the reliability of the results. The limitations of this study include the analysis was not adjusted for confounders such as comorbidities and severity of disease, the use of self-reported height when a measured height was not available (6% of the study sample), and that BMI is not in itself a perfect measure of obesity for those with atypical body fat/muscle rations, nor accounts for body fat distribution [33].

### 4.2. Implications for Clinical Practice

This study has confirmed a high prevalence of overweight and obesity in the hospitalized inpatient population, similar to population levels. It is evident malnutrition occurs across all weight classifications; however, the current focus of nutrition care is on both the identification and prevention of undernutrition, especially for patients who are in underweight or healthy weight classifications. There is an opportunity to highlight and support the identification of overnutrition for patients in all overweight and obese BMI categories, thus all forms of malnutrition and incorporating this into models of nutrition care. As modifiable risk factors, overweight and obesity need to be added to the nutrition risks included in current quality standards and incorporated into nutrition risk screening tools. Our study identified considerable variance in dietitians’ clinical decision-making for patients above their healthy weight range with overnutrition based on nutrition diagnosis, highlighting potential knowledge gaps that require further consideration for dietetic practice and management in the acute care setting.

## 5. Conclusions

This study has provided a comprehensive description of weight classifications and the prevalence of overweight and obesity in hospitalized inpatients across a multisite health service. The results demonstrate that overnutrition is highly prevalent amongst hospital inpatients and provide valuable clinical insight into the prevalence of overnutrition. These findings can be used to inform the design and delivery of dietetic services to an underserviced and nutritionally challenging group of patients to enhance patient and health outcomes.

## Figures and Tables

**Table 1:** General characteristics and weight classifications of the study sample by classification of weight status.

			Classification of Weight Status by BMI Category					*p* *
	All	Underweight	Normal	Overweight	Obese Class I	Obese Class II	Obese Class III	
		<18.5 kg/m^2^	18.5–24.9kg/m^2^	25–29.9 kg/m^2^	30–34.9 kg/m^2^	35–39.9 kg/m^2^	≥40 kg/m^2^	
Characteristics	513 (100%)	30 (5.8%)	189 (36.8%)	153 (29.8%)	79 (15.4%)	35 (6.8%)	27 (5.3%)	–
Age (years) Median (IQR)	73 (59–83)	89 (74–94)	78 (65–84)	73 (54–82)	69 (58–79)	72 (57–82)	64 (59–73)	<0.001
Sex (female) *n* (%)	255 (49.7%)	17 (56.7%)	91 (47.9%)	60 (39.5%)	45 (56.3%)	21 (61.8%)	21 (77.8%)	0.002
BMI (kg/m^2^) Median (IQR)	25.7 (22.2–30.4)	17.2 (16.4–18.2)	22.2 (20.7–23.7)	27.3 (25.7–28.5)	31.8 (30.7–33.2)	37.0 (35.9–38.2)	46.1 (43.8–50.6)	<0.001
Min-Max	13.9–78.1	13.9 –19.0	18.5–24.9	25.0–29.9	27.9–34.9	35.0–39.6	40.3–78.1	
Nutrition risk score								0.767
Not at risk *n* (%)	249 (68.8%)	12 (40%)	82 (43.4%)	73 (47.7%)	41 (51.9%)	23 (65.7%)	18 (66.7%)	
At risk *n* (%)	113 (31.2%)	11 (36.6%)	56 (29.7%)	32 (20.9%)	8 (10.1%)	3 (8.6%)	3 (11.1%)	
Incomplete *n* (%)	151 (29.4%)	7 (23.4%)	51 (27.0%)	48 (31.4%)	30 (38.0%)	8 (23.5%)	6 (22.2%)	
Malnutrition Diagnosis								<0.001
Yes *n* (%)	86 (16.8%)	21 (70%)	47 (24.9%)	14(9.2%)	1 (1.3%)	2 (5.7%)	1 (3.7%)	
Dietetic intervention								<0.001
Yes *n* (%)	191 (37.2%)	22 (73.3%)	77 (40.7%)	58 (37.9%)	19 (24.1%)	7 (20.0%)	8 (29.6%)	

(BMI: body mass index; IQR: interquartile range presented as 25th–75th percentile, * *p* was generated to compare associations between BMI category and demographic variables using Chi-square for categorical variables and Kruskal–Wallis for continuous variables.)

**Table 2:** General characteristics and weight classifications of the study sample according to the admitting clinical care program.

			Classification of Weight Status by BMI Category						*p* ^#^
		All	Underweight	Normal	Overweight	Obese Class I	Obese Class II	Obese Class III	
Program	Characteristics		<18.5 kg/m^2^	18.5–24.9 kg/m^2^	25–29.9 kg/m^2^	30–34.9 kg/m^2^	35–39.9 kg/m^2^	≥40 kg/m^2^	
Acute *	*n* (%)	330 (100%)	16 (4.8%)	107 (32.4%)	105 (31.8%)	60 (18.2%)	26 (7.9%)	16 (4.8%)	
64.3%	Age (years)								
	Median (IQR)	70 (55–81)	78 (69–89)	74 (59–83)	70 (48–82)	68 (57–77)	69 (48–73)	62 (48–70)	0.015
	Sex (female)								
	*n* (%)	147 (44.5%)	6 (4.1%)	45 (30.6%)	38 (25.9%)	33 (22.4%)	14 (9.5%)	11 (7.5%)	0.053
	BMI (kg/m^2^)								
	Median (IQR)	26.7 (23.1–31)	17.9 (17.1–18.3)	22.2 (20.8–23.8)	27.3 (25.7–28.7)	31.7 (30.7–32.9)	37.1 (36.1–38.3)	46.2 (41.4–51.9)	<0.001
Subacute *	*n* (%)	153 (100%)	14 (9.2%)	63(41.2%)	42 (27.5%)	17 (11.1%)	6 (3.9%)	11 (7.2%)	
29.8%	Age (years)								
	Median (IQR)	79 (65–86)	93 (89–96)	82 (71–86)	76 (64–84)	73 (58–82)	67 (60–81)	68 (60–82)	<0.001
	Sex (female)								
	*n* (%)	90 (58.8%)	11 (12.2%)	34 (37.8%)	20 (22.2%)	10 (11.1%)	5 (5.6%)	10 (11.1%)	0.047
	BMI (kg/m^2^)								
	Median (IQR)	25 (21.8–29.3)	16.45 (15.5–17.5)	22.3 (20.6–23.6)	27.6 (26.0–28.4)	32.7 (31.6–33.3)	36.9 (35.8–37.2)	46.0 (45.0–49.7)	<0.001
Transition Care *	*n* (%)	30 (100%)	0 (0.0%)	19 (63.3%)	6 (20.0%)	2 (6.7%)	3 (10.0%)	0 (0.0%)	
5.8%	Age (years)								0.730
	Median (IQR)	81 (73–86)	–	81 (72–86)	83 (80–86)	79 (68–89)	81 (72–81)	–	
	Sex (female)								0.355
	*n* (%)	18 (60%)	0 (0.0%)	12 (66.7%)	2 (11.1%)	2 (11.1%)	2 (11.1%)	0 (0.0%)	
	BMI (kg/m^2^)								
	Median (IQR)	23.5 (21–25.5)	–	21.4 (20.5–23.5)	25.4 (25.2–25.7)	30.0 (29.6–30.3)	35.0 (35.0–38.8)		<0.001

(BMI: body mass index; IQR: interquartile range presented as 25th–75th percentile; * percentage represents the proportion of the total study sample (*n* = 513), ^#^ *p* was generated to compare associations between BMI category and demographic variables using Chi-square for categorical variables and Kruskal–Wallis for continuous variables; analyses were conducted within each clinical program).

**Table 3:** Nutrition diagnosis of patients who had obesity and were receiving dietetic care.

Domain	Diagnosis	Number of Patients with Diagnosis
Clinical	Morbid obesity	3
	Well-nourished	7
	Severely malnourished	1
Intake	Inadequate intake	8
	Inadequate carbohydrate intake	1
	Adequate intake	1
	Excessive intake	1
Behavior	Nutrition knowledge deficit	1
	No diagnosis	11

**Table 4:** Summary of multiple regression model exploring the relationship between BMI, clinical characteristics, and other factors.

	Dependent Variable: BMI kgm^−2^		
Independent Variables:	Β	Sig	Contribution%
Age Sex	−0.217 −0.136	<0.001 0.000	4.12% 1.77%
Clinical Program	−0.042	0.239	0.23%
Malnutrition Screen (yes/no)	0.093	0.076	0.52%
Nutrition risk score	−0.165	0.020	1.56%
Malnutrition diagnosis (yes/no)	−0.277	<0.001	5.15%
Assessment and care by a Dietitian (yes/no)	0.014	0.775	

## Data Availability

Data is available on request due to ethical restrictions. The data is available on request from the corresponding author.

## Appendix A

**Table A1:** Comparison of proportion of study population vs. Victoria and Australia population norms by classification of weight status by BMI category, adults > 18 years. Adapted with permission from Australian Institute of Health and Welfare material Ref. [20]. 2022, Australian Institute of Health and Welfare Copyright ©.

Classification of Weight Status by BMI Category, Adults > 18 Years								
	Underweight	Normal	Total Underweight/ Normal	Overweight	Obese Class I	Obese Class II/III	Total Obese	Total Overweight/ Obese
	<18.5 kg/m^2^	18.5–24.9 kg/m^2^	<24.99 kg/m^2^	25–29.9 kg/m^2^	30–34.9 kg/m^2^	35.0 kg/m^2^	30.00 kg/m^2^	25.00 kg/m^2^ or More
Cohort %	5.8	36.8	42.9	29.8	15.4	12.1	27.5	57.1
Victoria [20]	1.0	30.6	31.7	36.6	20.5	11.3	31.8	68.3
National [20]	-	-	34.2	36.6	-	-	-	65.8
